# Myofunctional Speech Therapy for Facial Rejuvenation and Orofacial Function Improvement: A Systematic Review

**DOI:** 10.3390/jfmk9020099

**Published:** 2024-06-05

**Authors:** Luca Levrini, Giulia Baldelli, Chiara Castellani, Luigia Ricci, Claudia Paola Bruna Dellavia, Nicola Giannotta, Gaia Pellegrini, Stefano Saran

**Affiliations:** 1 Department of Human Sciences, Innovation and Territory, Post Graduate School in Orthodontics, University of Insubria, 21100 Varese, Italy; luca.levrini@uninsubria.it (L.L.); ngiannotta@studenti.uninsubria.it (N.G.); 2 Independent Researcher, 20131 Milan, Italy; giulia.baldelli6@gmail.com; 3 Independent Researcher, 35141 Padua, Italy; chiara.castellani25@gmail.com; 4 Independent Researcher, 04100 Latina, Italy; luigiariccilogo@gmail.com; 5 Department of Biomedical, Surgical, and Dental Sciences, University of Milan, 20100 Milan, Italy; claudia.dellavia@unimi.it (C.P.B.D.); gaia.pellegrini@unimi.it (G.P.)

**Keywords:** facial rejuvenation, skin aging, facial exercises, speech therapy, facial aesthetics, facial muscles

## Abstract

This review aims to reveal the effectiveness of myofunctional speech therapy on facial rejuvenation and/or improvement of orofacial function. A systematic review of four medical electronic databases (Medline, Google Scholar, SciELO, and LILACS) was conducted between January and March 2023. The research question was defined using the PICO model: Population (P): adult subjects with signs of physiological aging of facial skin. Intervention (I): aesthetic speech therapy (facial exercises and/or myofunctional therapy). Control (C): absence of treatment. Outcome (O): facial rejuvenation. Through the search process, a total of 472 potentially relevant articles were identified. A total of 21 studies were included in the review. Most of the studies required the participants to perform exercises learned during the weekly session on a daily basis. The subjects underwent an integrated treatment with facial exercises and worked on the stomatognathic functions for different durations. Many differences were found in the evaluation tools used to investigate the starting situation and the effects obtained following the treatment. At the diagnostic level, there was no concordance in the choice of the most appropriate scales and assessment tools, but great heterogeneity was observed. Indeed, forty-eight percent of the studies collected objective data through the use of various instruments (oral devices, electromyographs, cutometers, muscle ultrasound scans, and laser scans of the face). The observed improvements included not only a reduction in wrinkles and frown lines but also decreased muscle tension and slackness, enhanced facial symmetry and lip competence, improved skin elasticity, and restored stomatognathic function. These changes led to myofunctional restoration and facial rejuvenation, resulting in increased satisfaction with self-image and proprioception.

## 1. Introduction

Body image is a complex human phenomenon characterized by affective, cognitive, social, and physical aspects [1].

Aging is, moreover, considered a multidimensional, natural, gradual, and progressive process that cannot be stopped but, at most, delayed. Physiologically, anatomical, functional, physicochemical, and psychosocial changes occur, to varying degrees, caused by reduced function of organs and tissues, as well as the progressive loss of the individual’s ability to adapt to the environment. These changes are generated by intrinsic and extrinsic factors or by diseases or accidents, which, in general, increase the risk of vulnerability and disorders [2,3].

The characteristic signs of the aged face are the consequence of modifications to surface appearance, three-dimensional shapes, and expression. At the skin level, structural and functional changes cause progressive thinning, laxity, the appearance of fine rhytids, hyperpigmented or red areas, and alterations of the texture. At the deeper layers, changes in volumetric ratios between tissues and their overall deflation seem to be due to skeleton reshaping, bone remodeling, and the contraction of the deep and superficial facial fat compartments. During aging, these two adipose compartments undergo different volume changes, inducing a relative hypertrophy of the superficial one compared to the deep one. A loss of 18.4% was observed in the deep layers, while a reduction of 11.3% was found in the superficial ones. Furthermore, superficial fat is characterized by displacement relative to the underlying skeleton, while deep fat undergoes a gravitational descent due to the gradual laxity of the retaining ligaments [4,5].

The loss of the anatomical structures supporting the muscle insertion also seems to affect their pulley and lever systems, thus changing the muscle angle of action. Furthermore, the physiological age-related sarcopenia of muscles increases their tone in rest position and reduces their mass and the amplitude of movement. The motor action of elevator muscles, such as the zygomaticus major muscle, seems to be reduced, affecting the lifting capacity against the action of depressor muscles and of the downward gravitational pull. These structural and functional changes in muscles contribute to the aging appearance of the facial expression, both in static and dynamic conditions [5,6].

The stomatognathic system undergoes numerous changes related to the aging process, and each structural transformation results in a number of consequences at the functional level [6].

There is a growing interest in reducing facial aging through alternative approaches, such as acupuncture and facial acupressure, less invasive techniques usually performed by nonmedical professionals [7,8,9].

Although several treatments are available for facial rejuvenation, prevention against extrinsic aging remains the best approach and should be encouraged in all individuals [10].

Among the treatments that use less invasive methods to achieve a more harmonious and, above all, more natural result, aesthetic speech therapy should be counted. 

Aesthetic speech therapy is a new therapeutic approach that bases its theoretical foundations in the area of orofacial motility and is understood as “an area of speech therapy that deals with the prevention, evaluation, and rehabilitation of congenital or acquired disorders of the orofacial and cervical myofunctional system and its functions” [11,12,13,14]. This discipline aims to improve the functioning of the stomatognathic system through specific work on oral functions and, consequently, on all the muscles of this anatomical area, including the mimic muscles. This has made it possible to demonstrate that treatment of orofacial muscle imbalance generates not only functional but also aesthetic improvements in the face and neck. A deep knowledge of the structure and functioning of the stomatognathic apparatus must be accompanied by a good awareness of the physiological stages and factors responsible for aging [15].

The goals pursued by aesthetic speech therapy involve physiological reprogramming of impaired oral functions, rebalancing of the orofacial musculature, alleviating and/or eliminating excessive or inappropriate facial expressions, and achieving a balance between function and aesthetics. The achievable results will be facial muscle strengthening and/or relaxation, removing or attenuating wrinkles or expression marks.

The main goal of this systematic review was to investigate the effects of myofunctional speech therapy on facial rejuvenation and/or improvement of orofacial function.

## 2. Materials and Methods

A systematic review was conducted to determine how many studies exist in the field of cosmetic speech therapy. Specifically, the PICO (population, intervention, control, outcome) model was used to define the research question. In particular: P (population): adult subjects with signs of physiological aging of facial skin; I (intervention): aesthetic speech therapy (facial exercises and/or myofunctional therapy); C (control): absence of treatment; O (outcome): facial rejuvenation.

### 2.1. Search Strategy

To select literature appropriate for the project, a literature search was conducted between January and March 2023 in the following databases: Medline (via PubMed), Google Scholar, SciELO (Scientific Electronic Library Online), and LILACS.

The protocol of this research was registered in the publicly accessible PROSPERO (CRD42023447130), the primary database for registering systematic review protocols.

The reason for the decision to conduct a search in two South American databases (SciELO and LILACS) was not to exclude all studies conducted in Brazil and published in Portuguese in order not to narrow the field of research too much in an unknown speech therapy area. 

In addition, the field was not narrowed down in time in order to find the largest number of scientific studies conducted so far.

The keywords used in the search databases were facial rejuvenation, skin aging, facial exercises, myofunctional/speech therapy, facial aesthetics, and facial muscles.

Multiple search strings were used in the Medline database, with MESH used when available (Table S1: Search strings used in the different databases). 

### 2.2. Inclusion and Exclusion Criteria

The articles were selected according to the following inclusion criteria: (a) studies conducted on a healthy adult sample; (b) facial rejuvenation studies; (c) studies using facial exercises (with or without the use of devices) and/or myofunctional therapy to restore oral function.

On the other hand, the following were considered exclusion criteria: (a) systematic reviews and meta-analyses; (b) studies conducted on patients with head trauma, facial nerve palsy, or other pathological conditions; (c) studies on invasive methods of aesthetic medicine (surgery, Botox injection, filler, peeling, laser, acupuncture).

### 2.3. Data Collection and Extraction

The research was conducted according to the following review procedure: After identifying all records found in the databases, a duplicate elimination process was performed to proceed with the screening phase. For initial screening, the titles were screened, and the abstracts in each database were consulted. Finally, in the eligibility phase to select studies for the review, the full texts of the articles selected in the screening phase were analyzed. Several scales, including PEDro, STROBE, SCED, and JBI, were employed to assess the methodological quality of studies and to evaluate how effectively they addressed potential biases in their design, execution, and analysis.

The PEDro scale [16] was employed to evaluate the methodological quality of the experimental clinical trials included in the review. This scale comprises 11 items covering the domains of selection, performance, evidence, reporting, and allocation bases (excluding criterion 1, which pertains to the study’s internal validity). A study scoring 6 or higher is considered evidence-based 1 (with scores of 6–8 deemed good and 9–10 excellent), while a study scoring 5 or lower is considered evidence-based 2 (with scores of 4–5 deemed acceptable and below 4 poor) [17].

The STROBE statement [18] was used to evaluate the methodological quality of observational studies, containing 22 items that assess elements such as title, abstract, introduction, methods, results, discussion, and other relevant information.

The Single Case Experimental Design (SCED) scale [19], consisting of 11 items, was utilized to score case reports. Scores range from 0 to 10, with higher scores indicating better methodological quality.

Finally, the critical appraisal checklist developed by the Joanna Briggs Institute (JBI) [20] was applied to assess the quality of the case series, where negative responses negatively impact methodological quality.

## 3. Results

### 3.1. Selection of Studies

**Figure 1:**
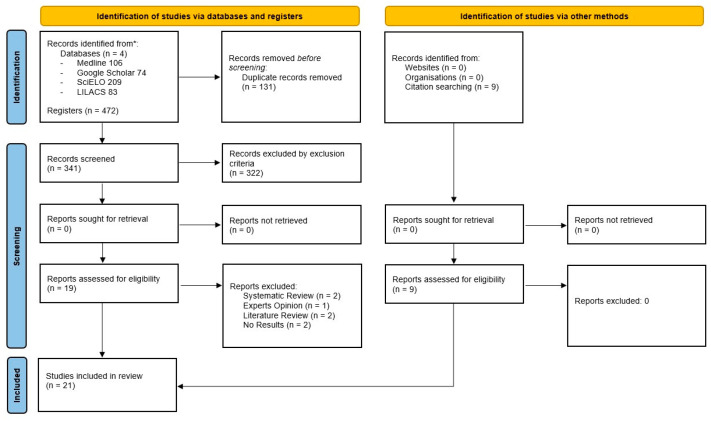
Flow chart: summary of the selection process according to the PRISMA guidelines.

Through the electronic database search process, a total of 472 potentially relevant articles were identified. A total of 131 duplicate articles was removed. There were 341 articles left to go through the screening process. From the latter, 322 articles were excluded based on the pre-established exclusion criteria identified by reading the title. Therefore, 19 articles remained to be analyzed by reading the abstract and full text. During this process, seven articles were excluded for the following reasons: (a) systematic reviews of the literature (*n* = 2); (b) expert opinions (*n* = 1); (c) literature reviews (*n* = 2); (d) absence of results (*n* = 2). Furthermore, the search was conducted within the bibliography of the selected studies, allowing the inclusion of nine articles. Therefore, a total of 21 studies were included in the review [2,7,21,22,23,24,25,26,27,28,29,30,31,32,33,34,35,36,37,38,39]. The whole selection process in the different stages is detailed in a PRISMA flowchart (Figure 1).

In Table 1, the list of articles selected and included in the systematic review is reported, pointing out the type of intervention, outcome measure, results, and efficacy.

### 3.2. Evaluation of Study Quality

The PEDro scale [16] was used to assess the methodological quality of the experimental clinical trials included in the review. This scale consists of 11 items related to the domains of selection, performance, evidence, reporting, and allocation bases (criterion 1, related to the internal validity of the study, is not included). A study with a score greater than or equal to 6 is considered evidence-based 1 (a score of 6–8 would be considered good, 9–10 excellent), and a study with a score of 5 or less is considered evidence-based 2 (a score of 4–5 is considered acceptable, <4 poor) [17] (Table S2: Quality of experimental studies measured by the PEDro scale). 

The STROBE statement was used to assess the methodological quality of observational studies. It contains a total of 22 items assessing items such as title, abstract, introduction, methods, results, discussion sections, and other information [18] (Table S3: Valuation of the quality of observational studies with STROBE).

The Single Case Scale Experimental Design (SCED) with 11 items was used to score the case reports. The score ranges from 0 to 10, with higher scores indicating higher methodological quality [19] (Table S4: Methodological quality of case reports assessed according to the SCED scale). 

Finally, the critical appraisal checklist developed by the Joanna Briggs Institute (JBI) was applied to assess the quality of the case series; negative responses have a negative impact on methodological quality [20] (Table S5: JBI critical appraisal checklist for case series).

### 3.3. Data Extraction

The total population of the 21 studies is 413 subjects. The studies were conducted on a total population of 64 male and 335 female subjects. In the study [21], the number of subjects divided by gender was not specified.

Analyzing the age of the total sample, an age between 18 and 87.3 years was identified (mean age: 52.65 ± 34.65 years); the study [33], however, only specified the mean age of 25 years. A total of 48% was conducted in Brazil (*n* = 10), 24% in Japan (*n* = 5), and about 10% in the United States (*n* = 2) and Korea (*n* = 2); however, two studies were conducted in Belgium (*n* = 1) and Malaysia (*n* = 1).

Regarding the language of publication, as explained in the section on “Search strategy”, the articles published in a language that is not English have been included to enlarge the research. Specifically, South America, which is particularly active in the field of orofacial motor activity research, was taken into consideration. 

Thus, 8 Portuguese-language studies (38%) and 13 English-language studies (62%) were analyzed. Most of the studies (38%) were published in the open-access online journal CEFAC, a Portuguese journal, with the aim of widespread scientific production on relevant topics in speech therapy, audiology, and related areas. The remaining articles have been published in multiple medical–scientific journals.

### 3.4. Study Design

The identification of the study design was, in some cases, complex as it was not always explicit, clear, and consistent with the methodology used.

Experimental studies were identified (62%); in these, the researchers, therefore, actively intervened by administering the treatment to be evaluated, modifying the starting situation. Only one observational study (5%) was identified, in particular case-control, in which no situation was manipulated but the difference between two groups of subjects was examined and analyzed; 10% is represented by case series, i.e., studies that observe and describe the effects of a treatment on a group of subjects sharing some characteristics; finally, two case reports were included (14%), which aim to describe individual clinical cases to illustrate and ascertain, in this case, the effectiveness of speech therapy treatment on facial aesthetics.

The experimental studies were designed differently, despite having a common goal, using a rigorous methodology. The studies with a higher level of scientific evidence are randomized controlled trials [7,35,39], in which subjects were randomly assigned to the experimental group, which received the treatment, or to the control group, which was not subjected to any type of therapy 

There are also non-randomized controlled studies [32,33] in which the randomized assignment of participants to the two groups was not used; the studies [33] and [39] divided the subjects into two experimental groups and a control group.

Furthermore, uncontrolled experimental studies were included, i.e., studies that verified the efficacy of the treatment in a single group of subjects, evaluating the difference between the pre- and post-treatment conditions [2,21,27,28,29,31,34,36,37]. The study [38] instead investigated the effect of the treatment on two experimental groups differentiated by different levels of education. Among the uncontrolled experimental studies, it is necessary to make a distinction between those that used a quantitative methodology [21,28,29,31,34,36,37,38], thus obtaining objective data to be analyzed statistically, and experimental studies that, instead, used a qualitative methodology, characterized by the analysis of photographs and answers to self-evaluation questionnaires [2,27]. This qualitative methodology was shared with the “case series” [23,24,26] and with the case reports [22,25].

What distinguishes the uncontrolled qualitative studies from the case series is the eventual personalization of the treatment; in the first case, the treatment was administered equally to all study participants; in the second case, however, the researchers subjected the subjects to an ad hoc treatment path based on their needs.

**Figure 2:**
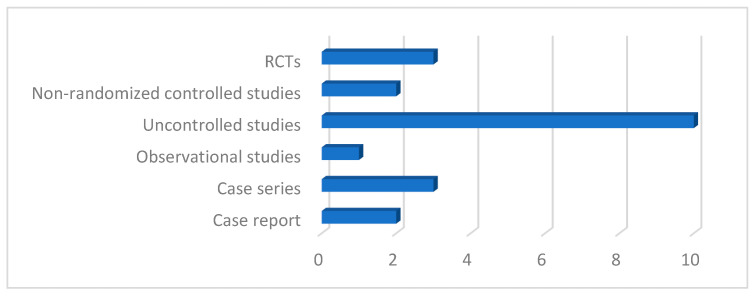
Distribution of study designs included in the review.

The only non-experimental study identified was the one conducted by Potter et al. [30], which did not administer any type of treatment but recruited two homogeneous groups of subjects, differentiated by the presence or absence of a certain characteristic (playing the trumpet); after the selection of the subjects, the differences between the cases and the controls were observed and statistically analyzed. Overall, six studies used a control group that did not receive treatment or was distinguished by the absence of a specific condition; thirteen studies investigated the efficacy of the treatment on a single experimental group; and two studies evaluated the efficacy of a treatment on a single case (Figure 2).

### 3.5. Objectives and Type of Intervention

The main objective of all research was to investigate the effects of myofunctional speech therapy on facial rejuvenation and/or on the improvement of orofacial functions. The study [38] conducted by Souza and Porto (2022), on the other hand, analyzed the relationship between the level of education and the quality of life of women subjected to myofunctional treatment of facial aesthetics.

The speech therapy interventions in which the subjects were involved and whose effectiveness was evaluated are divided into facial exercises (*n* = 9), facial exercises associated with using a device (*n* = 7), and facial exercises associated with work on stomatognathic functions (*n* = 4).

**Figure 3:**
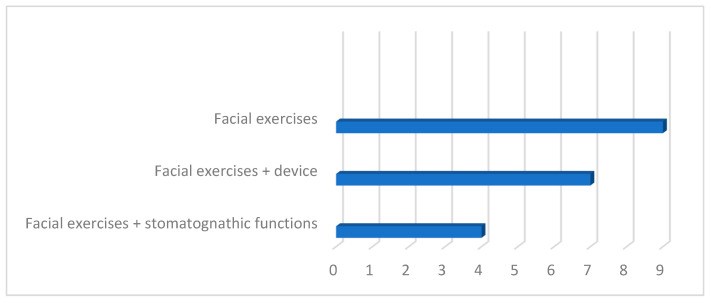
Distribution of searches based on the type of treatment proposed.

The proposed facial exercises have aimed to train different types of muscles, and not all studies have taken into consideration the importance of stomatognathic functions for better muscle balance and greater harmony on an aesthetic level (Figure 3).

Lana e Silva et al. [23], for example, studied the effectiveness of two speech therapy techniques for facial aesthetics on the orbicularis oculi muscle. In particular, five repetitions of massages on the orbicularis muscle of the eyes were applied on one side of the face, while five repetitions of an isometric facial exercise for the eye muscles were proposed on the other side. This study, therefore, did not consider any oral function; however, it obtained positive results on the reduction in expression lines, with no differences between the techniques used.

Some studies wanted to analyze the effectiveness of training the orbicularis labialis muscle associated with the use of an oral device on the improvement of various oral functions in which the lips themselves are involved (swallowing, articulation, breathing, chewing) [28,29,32,33,34,35].

These studies have also shown positive effects, both functionally and aesthetically.

Studies that integrated facial exercises and work on stomatognathic functions into the treatment protocol obtained good results in improving the aesthetics of the face [21,24,38,39]. The study of Ferreira et al. (2022) [39] investigated, through two experimental groups, the effects of two therapeutic strategies on the electrical activity of the suprahyoid muscles and the self-perception of aesthetic changes in the submandibular region. Specifically, the difference between performing the single lingual pressure exercise on the retroincisal papilla and the combination of the latter with functional swallowing training was studied. The results underlined that both strategies increased the electrical activity of the suprahyoid musculature but did not have a significant impact on the self-perception of visual and muscular improvements in the submandibular region. Some research [21,24,38], on the other hand, investigated the effectiveness of speech therapy applied to facial aesthetics through a single protocol performed by a single group of subjects, using both facial and stomatognathic exercises (masticatory training, swallowing, and articulation).

Exclusive use of facial exercises was verified in various studies; the muscle training proposed in these studies was applied in all cases (100% of the studies) on all parts of the face [2,7,23,25,26,27,31,38]. One study [37] evaluated the effectiveness of facial exercises using the Pao facial rejuvenation device. The latter differs from studies on the lip muscles with the use of oral devices since it involves training the muscles of the middle third and lower third, muscles considered by the authors to be more identifiable for pre- and post-treatment ultrasound evaluation.

The only study that did not investigate the effectiveness of a therapeutic protocol but wanted to examine how playing the trumpet can lead to an increase in the strength and resistance of the muscles of the lips, cheeks, and tongue is the one conducted by Potter [30]. In particular, trumpet players have been shown to produce and manipulate sound through that instrument by articulating the lips, cheeks, and tongue to direct the airflow and create an appropriate embouchure [40]. To verify the increase in strength and resistance of the lips, cheeks, and tongue, adult trumpeters who practiced at least 6 h a week and had an experience of at least 8 years were compared with subjects who did not play the trumpet. The results showed that trumpet players had greater lip resistance but not greater lip strength, increased cheek strength but not increased cheek strength, and no differences in tongue strength and resistance compared to controls.

The facial exercises proposed in all studies were isometric exercises, with the aim of increasing muscle strength and endurance. In some research, this type of exercise has been associated with isotonic exercises, manipulations, the use of devices, and specific work on oral functions.

### 3.6. Duration of Treatment Programs

By analyzing the duration of each treatment in terms of the number of weeks, it was possible to observe how much variability there is between the different types of treatment and also within them. Furthermore, most of the studies required the participants to perform the exercises learned during the weekly session on a daily basis.

Studies of oral orbicularis muscle training associated with oral device use have, in most cases, a duration of 4 weeks [29,32,33,34,35]; one examined lip-lock strength over 7 days [35]; another work investigated the effect of lip exercise with the oral appliance on improving lip closure strength and lingual elevation, as well as skin elasticity, over 14 and 24 weeks (6 months) [28].

The researchers guided the subjects during the integrated treatment with facial exercises and worked on the stomatognathic functions, in some cases, for a duration of 8 weeks [32], 10 weeks [24,38], and 16 weeks [21]. Ferreira et al. (2022) did not show significant results from an aesthetic point of view.

The duration of the facial exercise programs was predominantly 8 weeks [7,22,25,27,31]; a study proposed a 4-week treatment [27]; a longer 12-week protocol was considered in 2 studies [2,26]; moreover, the exercise program that lasted the longest was the one proposed in the research of Alam et al. (2018) [36], with 20 weeks of treatment.

The study, which evaluated the effectiveness of facial exercises with the use of the Pao device, proposed eight training sessions, falling within the time range seen so far [37].

**Figure 4:**
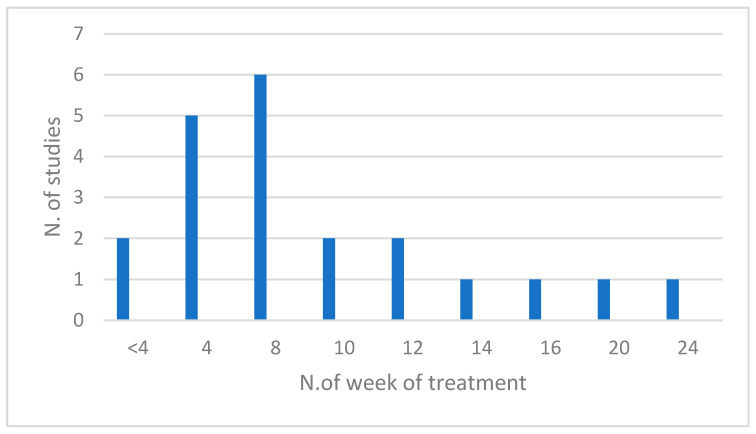
Distribution of searches by number of weeks of treatment.

Finally, the research of Lana e Silva et al. [23] focused on the exclusive treatment of the orbicularis oculi muscle with a duration of 20 days (Figure 4).

### 3.7. Evaluation Tools and Outcomes

Finally, many differences were found in the evaluation tools used to investigate the starting situation and the effects obtained following the treatment. Some studies used qualitative, others quantitative, assessment, and outcome measures.

The first studies, conducted from 2002 to 2012, reported mostly subjectively assessed results with self-perception questionnaires on the changes obtained with the treatment, questionnaires filled in by other people, researchers’ observations on the changes obtained, and photographic analyses before and after the treatment itself. Some authors have verified the effectiveness of the treatment using anthropometric measurements of the face, using not only descriptive and subjective but also quantitative measures, statistically analyzing the measurements obtained with the caliper, and analyzing the distance between the nasolabial fold and the tragus before and after treatment [21,22,24]. 

Since 2013, more rigorous studies have been conducted from a methodological point of view. In particular, tools capable of providing data and objective measurements have been applied, such as oral devices capable of quantitatively measuring labial and lingual strength and resistance [28,29,32,33,34,35]; the electromyograph [31,39]; the cutometer to evaluate elasticity as a mechanical property of the skin [28,31]; and muscle ultrasound scans and laser scans of the face [29]. These tools have often been combined with self-assessment and self-perception questionnaires [7,36,39], with the analysis of photographic documentation using validated quantitative VAS scales (e.g., MCFAP—Merz-Carruthers Facial Aging Photoscales; WSRS—Wrinkle Severity Rating Scale) [7,36,38]. The analysis of quality of life, the muscle improvement protocol in speech therapy for facial aesthetics (PAMFEF), the assessment of facial aesthetics, and photographic documentation were used as outcome measures in the socio-demographic questionnaire.

It can, therefore, be observed how the choice of evaluation and outcome measures were different according to the different authors based on the objective of the study.

## 4. Discussion

Aging, understood as a physiological process of the human body, is reflected in the skin in the form of spots, wrinkles, and expression lines.

The request for a rejuvenated appearance has been a theme pursued by men and women since ancient times. This constant search has allowed the development of numerous methods that can meet the needs and demands of many people. Among the less invasive techniques, it is possible to include aesthetic speech therapy, a type of treatment for orofacial motility that has been allowed to be used for aesthetic purposes since 2008. This intervention “aims to assess, prevent, and balance facial and/or cervical mimic musculature and orofacial functions, seeking symmetry and harmony of the involved structures, movement, and expression, resulting in aesthetic improvement” [13].

Therefore, this systematic review was conducted with the aim of investigating the effectiveness of aesthetic speech therapy for facial rejuvenation.

The analysis of the studies showed a greater demand and participation of the female sex (84%), which is confirmed by the annual statistics of the International Society of Aesthetic Plastic Surgery. For example, in the 2021 report on facial surgical procedures, facelifts were performed by women in 83% of cases; the same figure is found for non-surgical cosmetic procedures for facial rejuvenation, which were performed by women in 87% of cases.

Regarding the age group studied, the studies were performed on subjects with an overall average age of 52.65 years, an average age at which both men and women experience decreased hormone production associated with a decrease in synthesized collagen [41,42], as well as physiological atrophy of adipose tissue [43] and even of the mimic muscles of the face and neck [42].

At the diagnostic level, there was no concordance in the choice of the most appropriate scales and assessment tools, but great heterogeneity was observed. The assessment of the first studies conducted in this area was conducted by observation and palpation of the muscles in conjunction with photographic documentation that could be compared after treatment. On the other hand, forty-eight percent of the studies collected objective data through the use of various instruments (oral devices, electromyographs, cutometers, muscle ultrasound scans, and laser scans of the face).

This made it possible to provide objective and statistically relevant results and to circumvent the subjectivity of self-perception and satisfaction questionnaires.

Another aspect to consider is the low interest in expanding and complementing the aesthetic assessment of the face with an in-depth study of stomatognathic functions, which were analyzed in only 24% of the studies [1,21,24,26,30].

It is important to treat orofacial muscle imbalances comprehensively and to include oral function in the treatment protocol without being limited exclusively to the elimination of facial wrinkles or blemishes. In this review, only 19% of studies planned to work on one or more stomatognathic functions accompanied by facial exercises targeting specific facial muscles.

Regarding treatment protocols, 52% of studies suggested speech therapy for all facial thirds, from upper to lower; one study focused only on the orbicularis muscle of the eyes [31]. Twenty-nine percent of studies focused on the orbicularis muscle of the lips, the latter having a direct relationship with oral breathing [28,29,32,33,34,35]. A study [29], on the other hand, showed treatment results in the middle and lower thirds of the face; finally, only one article [20] analyzed the activity of the suprahyoid muscles involved in many oral functions, such as swallowing, chewing, articulation, and breathing [44].

Regarding the duration of the proposed treatment programs, a strong heterogeneity was found, with a time range mainly between 4 and 12 weeks. This finding is confirmed in the literature by studies that have investigated the effectiveness of myofunctional therapy in the presence of dysfunctional swallowing [45], obstructive sleep apnea [46,47], or altered lingual frenulum [48,49,50,51], reporting an average duration of 2–3 months. In some cases, the duration and intensity of cosmetic speech therapy were insufficient to achieve positive results, which negatively correlated with efficacy [7,39].

Some scientific studies [26,34] have considered the type of muscle fibers trained as potentially relevant information on which the effectiveness of the treatment itself could depend. Regarding the anatomical–histological aspect of facial muscles, there are few studies in the literature. A rather dated article [52] showed great variability in the fiber composition of the complex facial muscle system, which, however, is reflected in highly differentiated functions such as tone, expressive power, and synergistic activity of the muscles in performing oral functions. In particular, a high percentage of type I fibers (fibers with reduced contraction speed and force but high fatigue resistance) was found in the postural muscles of the face (M. buccinator, M. frontalis, M. corrugator of the eyebrows, and M. depressor of the lower lip). In contrast, type II fibers (fibers with high contraction speed and force but low fatigue resistance) were found in phasic muscles such as the orbicularis muscle of the eye and the nasal muscle. The rest of the musculature seems to be characterized by intermediate fibers, namely, a mixed composition of phasic and tonic fibers [52].

In a report on Glosso-postural syndrome [53], it was considered that in the presence of muscular imbalance, usually the phasic muscles (type I fibers) tend to weaken while the tonic postural muscles (type II fibers) tend to tense. Therefore, the former may need to be trained, whereas the latter may need to be relaxed. Therefore, knowledge of the anatomical–histological characteristics of the muscles to be treated may be an important aspect of the success of the treatment itself.

In general, 90% of the studies included in the review reported positive results either with the use of facial exercises alone or with the use of oral appliances to perform myofunctional exercises, as well as with protocols that included facial exercises and work to restore stomatognathic function.

In most of the studies, the efficacy was evaluated through a self-perception questionnaire, photographic and video documentation, anthropometric measurements, manual palpation, electromyographs, and oral devices.

It is also right to affirm that there are other reviews that have discordant conclusions regarding the efficacy of facial exercises in order to achieve significant rejuvenation, but they report that there is low evidence [11,12]. 

John Van Borsel et al. [11] have stated that the current evidence is inadequate to ascertain the effectiveness of facial exercises for facial rejuvenation. To draw definitive conclusions, evidence from large randomized controlled trials is necessary.

Hyoung Won Lim [12] affirmed that, despite these positive outcomes for facial rejuvenation and muscle strengthening, the level of evidence was low. Consequently, future research must investigate the effects of facial exercise through rigorously controlled experiments with adequate sample sizes to enhance the level of evidence.

### Limitations

Overall, we must mention some limitations of this review, including the limited number of randomized, controlled experimental studies. In addition, of the 21 studies analyzed, only six included a control group [7,30,32,33,35,39]. Forty-eight percent of the research studies examined the efficacy of the intervention in only one subject group; the remaining studies (*n* = 5) were case reports and case series. The studies were conducted with small sample sizes; the study with the largest sample size is [33] and has a total of 76 subjects. Another limit that has to be considered is the usage of the self-questionnaire, which can be misunderstood. A questionnaire was adopted in nine articles included in the review [2,7,23,26,27,36,38,39]. The photographic evaluation can be biased when it is applied without an objective measurement, as in articles [2,7,23,25,26,27,36,38]. The ages of the patients included in the articles selected were quite different. In fact, one study [35] included patients over eighty years old, and another one [34] involved patients who were under twenty-six years old. This aspect can limit our study because of the different biological ages. Other studies just included Pat. Finally, only four studies included oral function analysis in the treatment protocol. 

## 5. Conclusions

The results of the present systematic review show positive effects on facial aesthetic rejuvenation and muscle enhancement following aesthetic speech therapy treatment. The improvements observed include not only a reduction in wrinkles and frown lines but also a reduction in muscle tension and slackness, the achievement of greater facial symmetry and lip competence, the improvement of skin elasticity, and the restoration of stomatognathic function. These changes resulted in myofunctional restoration and facial rejuvenation, increasing satisfaction with self-image and proprioception.

With these positive results, the methodological quality and the limited number of studies performed must be considered. Therefore, more studies with a larger sample and differentiation of the type of speech intervention are recommended to help speech therapists understand the most effective approach to improving facial aesthetics.

## Figures and Tables

**Table 1:** Summary of included studies.

Author, Year	Study Design	Participants	Type of Intervention	Outcome Measure	Results	Efficacy
Takacs et al., 2002 [2]	Uncontrolled experimental study	8 adults (age: 31–66) -6 Females -2 Males	Facial exercises (12 weekly sessions) - isometric exercises 3/3 of the face	Self-perception questionnaire Photographic documentation	Reduction in wrinkles, facial expressions, and flaccidity in all individuals, with variations in the degree and location of improvement.	YES
Paes et al., 2007 [21]	Uncontrolled experimental study	10 adults (33–63 years old)	Facial exercises + work on stomatognathic functions (16 sessions weekly) -isometric exercises -isotonic exercises -isokinetic exercises -massages -facial manipulations -stomatognathic functional balance (including chewing training)	Self-perception questionnaire Measurement of distance between furrow nasolabial and tragus (pre and post) Photographic documentation	Significant reduction and remarkable balance between measurements of the projection from the nasogenian sulcus to the tragus on both sides. The exercises that caused the most positive results were those on the cheeks and mouth. -6/10 subjects reported a sensation of muscle lightness; -4/10 subjects reported rest and rejuvenation.	YES
Mattia et al., 2008 [22]	Case report	1 Female (56 anni)	Facial exercises (8 weekly sessions) -Change in posture -Stretching of muscles and reduction in tension -Cervical strain -Muscle relaxation -Facial stretching -Heating -Manipulation maneuvers (~20 min) -Facial exercises (~20 min) -General advice (related to bilateral chewing, hydration)	Anthropometric measurements Photographic documentation	Reduction in wrinkles, furrows, and sagging of the facial skin. Increased blood circulation in the face. Improvement of facial symmetry.	YES
Lana e Silva et al., 2010 [23]	Case series	4 females (age: 40–51)	Facial exercises for the orbicularis oculi (20 days) -left hemiface exercise -right hemiface massage	Self-perception questionnaire Photographic documentation	Differences were observed in all four patients. All patients experienced improvement. There were no differences between the techniques.	YES
Matos et al., 2010 [24]	Case series	4 females (age: 55–87)	Facial exercises + work on stomatognathic functions (10 sessions weekly)	Photographic documentation Anthropometric measurements	Decreased measurement from the labial commissure to the tragus; improvement of facial symmetry; vermilion increase in upper lip in 2/4 subjects. Wrinkle reduction, reduced muscle tension, greater eye-opening, improvement of mandibular contour, and reduction in flaccidity. Mimic improvement in chewing, swallowing, and articulation functions. Lateralization of food during chewing.	YES
Santos e Ferraz 2011 [25]	Case report	1 Female (age: 47)	Facial exercises (8 weekly session) -masseter stretching -manipulation -isometric exercises	Photographic documentation	Patient: -Improved feeling of well-being -Reduction in wrinkles and expression lines. Authors: -Improvement of facial symmetry and functions related to mandibular biomechanics.	YES
Frazao e Manzi et al., 2012 [26]	Case series	3 females (age: 41–49)	Facial exercises (12 Weekly sessions) -isometric exercises -isotonic exercises -stretching -massages	Satisfaction questionnaire Photographic and video documentation evaluated by authors	All three patients declared themselves satisfied with the treatment. Reduction in signs of aging. The aesthetic improvement in all cases was obtained by the reorganization of the facial muscle dynamics in the different oral functions.	YES
Arizola et al., 2012 [27]	Uncontrolled experimental study	11 females (age: 40–50)	Facial exercises (5 weekly sessions) -isometric exercises -isotonic exercises -manipulation -stretching	Self-perception and satisfaction questionnaire Photographic documentation	Changes perceived by all subjects; changes perceived by third parties on five subjects; satisfaction with facial appearance increased significantly. The agreement between specialists (speech therapists, dermatologists, and facial surgeons) was insufficient. In some aspects, changes were perceived.	YES
De Vos et al., 2013 [7]	RCTs	18 females (age: 39–60) -9 subjects (experimental group.) - 9 Subjects (control group)	Facial exercises (7 weekly sessions) -4 isometric exercises	Self-assessment questionnaire Photographic documentation + VAS	The exercises used in this study did not result in facial rejuvenation. Difference was found in the upper lip where the orbicularis muscle was subjected to a greater intensity (recruitment in two exercises).	NO
Ibrahim et al., 2013 [28]	Uncontrolled experimental study	13 healthy females (age: 41.3–48.1) (sleep apnea, snoring, mouth breathing)	Facial exercises + oral device 14 sessions weekly (17 subjects) 24 weekly sessions (13 subjects)	Lip/Decum oral device to evaluate the lip closure strength (LCS) and the lingual elevation force (TES). Cutometer to evaluate skin elasticity (SE).	Orofacial exercise performed for 14 weeks helps improve CSF, TES, and SE. The exercise also improved facial skin elasticity, indirectly nourishing facial tissue. Furthermore, extending the orofacial exercise period to 24 weeks significantly improved CSF and TES.	YES
Ohtsuka et al., 2015 [29]	Uncontrolled experimental study	18 healthy subjects with lip incompetence (age: 22.5–27.5) -12 males -6 females	Facial exercises + oral device (4 sessions weekly) -lip resistance training	Lip seal ratio -lip contact sensor -electric recording device Strength and endurance of the orbicularis labia -traction plates	The training of lip strength increases orbicularis oris strength and sealed lip ratio for subjects without any malocclusion and without oral breathing.	YES
Potter et al., 2015 [30]	Analytical observational case-control study	33 adults (age: 18–74) -16 trumpet players -16 subjects (control group9	Comparison of Differences in lip, cheek, and tongue strength and endurance between adult trumpet players who practice at least 6 h per week and non-trumpet players	IOPI	The trumpeters had greater cheek strength and greater lip strength compared to controls. There was no difference in the strength and resistance of the sound.	YES
Kim et al., 2016 [31]	Uncontrolled experimental study	16 females (age: 35–58)	Facial exercises (8 sessions weekly) -isometric exercises against resistance -neck and lip stretching	Superficial electromyograph Cutometer (evaluation of mechanical properties of the skin)	The results of the study mean that the skin becomes firmer and more elastic. Furthermore, skin fatigue and the ability to return to the original position are improved.	YES
Kaede et al., 2016 [32]	Controlled experimental study	20 subjects (age: 24–30) -10 (sperimental group: 5 M e 5 F -10 (control group): 5 M e 5 F	Facial exercises + oral device (4 sessions weekly) - improve lip closing strength	Multidirectional measurement of the force of lip closure	The closing force of the lips was significantly increased in the experimental group in the upward and downward directions compared to the control group.	YES
Fujiwara et al., 2016 [33]	Controlled experimental study not randomized	76 females (average age 25 years old) -10 (controlled group) -32 (experimental group trained with 200 g of water) -34 (experimental group trained with 400 g of water)	Facial exercises + oral device (7 days) - lip strength training	Measurement of multidirectional lip closing force (DLCF) with a device	The closing force of the lips increased following repetitions, even if in a short training period and with soft loads. These findings suggest that soft daily strength training, such as repeated lip locking, may be effective in increasing lip function.	YES
Yoshizawa et al., 2016 [34]	Uncontrolled experimental study	20 healthy subjects (age: 21.3–25.9) with lip incompetence -10 males -10 females	Facial exercises + oral device (4 sessions weekly) -hypoxic training of the orbicularis labial muscle to improve lip incompetence)	-Lip tightness ratio -lip contact sensor -electric recording device Tensile strength of the orbicularis labia: -measured by force needed to push the plate out of the oral vestibule	Hypoxic training of the lips increases the ratio of sealed lips and is, therefore, effective in improving the incompetence of the lips. The lip seal ratio decreased slightly but was maintained for 8 weeks after the end of training.	YES
Takamoto et al., 2017 [35]	RCTs	20 old subjects (85.3–87.3 age) -10 (experimental group: 2 males e 8 female) -10 (control group: 10 females)	Facial exercises + oral device (4 weekly sessions) -lip lock training	Maximum closing force of lip -digital measuring device (Lip De Cum) Eating behavior -digital video cameras Activity-rest rhythm -three-axis accelerometer Cerebral hemodynamic activity during the lip closing movement -near-infrared spectroscopy (NIRS)	The training of lip closure improved eating disorders, decreased daytime sleep, and activated the prefrontal cortex in elderly adults. These findings suggest that lip-lock training improves not only activities of daily living but also determines an increase in brain functions, suggesting its utility in older individuals with impaired oral/eating and cognitive functions.	YES
Alam et al., 2018 [36]	Uncontrolled experimental study	16 females (40–65 years old)	Facial exercises (20 sessions per week)	Satisfaction questionnaire Photographic documentation + MCFAP scale (validated scale)	Blind assessments Validated photographic scales showed significant improvement in upper and lower cheek fullness. Participants were highly satisfied, noting significant improvement in 18/20 facial features.	YES
Hwang et al., 2018 [37]	Uncontrolled experimental study	50 females (age: 30–63)	Facial exercises + device (8 sessions weekly)	Ultrasound scans -facial muscle thickness -cross-sectional area (CSA) Facial surface laser scanning -area -volume Wrinkle Severity Rating Scale (WSRS) Facial Visual Scale (FVS) (wrinkles and sagging of the chin line)	Changes in the WSRS scale and the FVS scale. Muscle thickness Facial and musculature cross-sectional areas increased, while facial surface distances, surface areas, and volumes decreased. They have not changed significantly: - the muscle thickness of the left side of the superior labrum - the thickness of the right side of the orbicularis oris - the distances of the surface of the face in the upper transverse part	YES
Souza e Porto et al., 2022 [38]	Uncontrolled experimental study	44 women (age: 50–56) divided according to the level of education -22 (group 1): finished middle and high school -22 (group 2): higher level of education	Facial exercises + work on stomatognathic functions (14 sessions weekly) -facial stretching -massages -isometric exercises -stomatognathic exercises (chewing, swallowing and/or articulation) -increased awareness of repeated facial expressions	SF-36 Social questionnaire- demographic based on the WHOQOL PAMFEF (Muscle Improvement Program Protocol in Facial Aesthetic Speech Therapy) Facial aesthetic evaluation protocol (Pierotti) Photographic documentation	Myofunctional therapy changed/improved muscle tension, chewing, swallowing, and articulation in both groups of women, with a statistically significant difference in pre- and post-treatment results. After myofunctional treatment, statistically significant changes were observed in the forehead, glabella, and periorbital wrinkles of both groups.	YES
Ferreira et al., 2022 [39]	RCTs	27 females (age: 30–78) -9 (group 1): isometric exercises -9 (group 2): isometric exercises + swallowing -9 (group 3): no exercise	Facial exercises (isometrics to strengthen the suprahyoid muscles by pressing the tip of the tongue against the hard palate at the level of the papilla): Group 1 Facial exercise (isometric) + work on oral swallowing function: Group 2 No exercise: Group 3 (8 sessions weekly)	Electromyographic evaluation Self-perception questionnaire on visual and muscular changes Weight measurement	The two strategies—lingual pressure exercises against the incisive papilla and the same exercise in combination with functional swallowing training—were equally effective in increasing the recruitment of the suprahyoid muscles, which was verified with surface electromyography. However, they did not have a significant impact on self-perception of visual and muscular improvements in the submandibular region.	NO

## Data Availability

The corresponding author can share the data upon request.

## Supplementary Materials

The following supporting information can be downloaded at: https://www.mdpi.com/article/10.3390/jfmk9020099/s1, Table S1. Search strings used in the different databases, Table S2. Quality of experimental studies measured by the PEDro scale, Table S3. Valuation of the quality of observational studies with STROBE, Table S4. Methodological quality of case reports assessed according to the SCED scale, Table S5. JBI Critical Appraisal Checklist for Case Series.
