# Supplementary material for: Myofunctional Speech Therapy for Facial Rejuvenation and Orofacial Function Improvement: A Systematic Review
Source: J Funct Morphol Kinesiol. 2024 Jun 5;9(2):99. doi: 10.3390/jfmk9020099 (PMC11204933; doi:10.3390/jfmk9020099)
Supplement: Supplementary file 1 [file jfmk-09-00099-s001.zip › jfmk-2974537-supplementary.pdf]

Table S1. Search strings used in the different databases.

	Search Strategy
PubMed	((("Skin Aging"[Mesh]) OR (skin aging)) OR (skin ageing)) AND ((((((("Myofunctional Therapy"[Mesh]) OR (myofunctional therapy)) OR (orofacial myofunctional therapy)) OR (oral myofunctional therapy)) OR (facial exercise*)) OR (speech therapy)) OR ("Speech Therapy"[Mesh])): 4 results<br><br>(((("Skin Aging"[Mesh]) OR (skin aging)) OR (skin ageing)) AND (facial exercise*)) AND ((("Rejuvenation"[Mesh]) OR (rejuvenation)) OR (facial rejuvenation)): 6 results<br>((("Skin Aging"[Mesh]) OR (skin aging)) OR (skin ageing)) AND (facial exercise*): 29 results<br><br>(facial exercise*) AND ((("Rejuvenation"[Mesh]) OR (rejuvenation)) OR (facial rejuvenation)): 16 results<br><br>((((((("Myofunctional Therapy"[Mesh]) OR (myofunctional therapy)) OR (orofacial myofunctional therapy)) OR (oral myofunctional therapy)) OR (facial exercise*)) OR (speech therapy)) OR ("Speech Therapy"[Mesh])) AND ((("Rejuvenation"[Mesh]) OR (rejuvenation)) OR (facial rejuvenation)): 17 results<br><br>((("Skin Aging"[Mesh]) OR (skin aging)) OR (skin ageing)) AND ((((((("Myofunctional Therapy"[Mesh]) OR (myofunctional therapy)) OR (orofacial myofunctional therapy)) OR (oral myofunctional therapy)) OR (facial exercise*)) OR (speech therapy)) OR ("Speech Therapy"[Mesh])): 34 results<br><br>(("Speech Therapy"[Mesh]) OR (speech therapy)) AND ((("Rejuvenation"[Mesh]) OR (rejuvenation)) OR (facial rejuvenation)): 3 results
Google Schoolar	"skin aging" "myofunctional therapy"|"orofacial myofunctional therapy"|"oral myofunctional therapy"|"facial exercises"|"speech therapy" "facial rejuvenation"|"rejuvenation": 27 results<br>"skin aging" "facial exercises" "facial rejuvenation"|"rejuvenation": 22 results<br>"facial exercises" "facial rejuvenation": 45 results<br>"skin aging" "facial exercises" : 26 results<br>“myofunctional therapy” “facial rejuvenation”: 13 results
SciELO	facial exercises (in all indices): 18 results<br>facial aesthetics (in all indices): 80 results<br>facial esthetics (in all indices): 97 results<br>(speech therapy) AND (facial esthetics): 6 results<br>(facial exercises) AND (facial esthetics): 4 results<br>(myofunctional therapy) AND (facial esthetics): 4 results
LILACS	Facial exercises (in all indices): 57 results<br>(facial exercises) AND (facial rejuvenation): 11 results<br>(myofunctional therapy) AND (facial rejuvenation): 1 result<br>(speech therapy) AND (facial aesthetics): 14 results

Table S2. Quality of experimental studies measured by the PEDro scale.

Author (year)	Score	Level of evidence
Takacs et al. (2002)	1/10	SCARCE
Paes et al. (2007)	3/10	SCARCE
Arizola et al. (2012)	3/10	SCARCE
De Vos et al. (2013)	7 / 10	GOOD
Ibrahim et al. (2013)	3 / 10	SCARCE
Ohtsuka et al. (2015)	3 / 10	SCARCE
Kaede et al. (2015)	4 / 10	ACCEPTABLE
Fujiwara et al. (2016)	4 / 10	ACCEPTABLE
Kim et al. (2016)	3 / 10	SCARCE
Yoshizawa et al. (2016)	3 / 10	SCARCE
Alam et al. (2018)	2 / 10	SCARCE
Hwang et al. (2018)	3 / 10	SCARCE
Takamoto et al. (2018)	7 / 10	GOOD
Ferreira et al. (2022)	8/10	GOOD
Souza e Porto (2022)	5/10	ACCEPTABLE

Table S3. Valuation of the quality of observational studies with STROBE.

Evaluation section	Item	Potter et al. (2015)
Title and abstract	1	X
I: premises/rational	2	X
I: aims	3	X
M: study design	4	X
M: framework	5	X
M: subjects	6	X
M: variables	7	X
M: data source/ detection	8	X
M: biases	9	
M: sample size	10	
M: qualitative variables	11	
M: statistical methods	12	X
R: subjects	13	
R: descriptive data	14	
R: results	15	X
R: main results	16	X

R: other analysis	17	X
D: main results	18	X
D: limits	19	X
D: interpretation	20	X
R: generalizability	21	
R: other information (funding)	22	
I: Introduction; M: Material and Methods; R: Results		

Table S4. Methodological quality of case reports assessed according to the SCED scale.

Authors, (year)	Item 1	Item 2	Item 3	Item 4	Item 5	Item 6	Item 7	Item 8	Item 9	Item 10	Item 11	Total
**Mattia et al. (2008)**	1	1	1	1	1	0	0	0	0	0	0	**5/10**
**Santos e Ferraz (2011)**	1	1	1	1	1	0	0	0	0	0	0	**5/10**

Table S5. JBI Critical Appraisal Checklist for Case Series.

**Lana e Silva et al. (2010)**				
**Questions**	**Yes**	**No**	**Unclear**	**Not applicable**
1. Were there clear criteria for inclusion in the case series?	X			
2. Was the condition measured in a standard, reliable way for all participants included in the case series?	X			
3. Were valid methods used for identification of the condition for all participants included in the case series?		X		
4. Did the case series have consecutive inclusion of participants?		X		
5. Did the case series have complete inclusion of participants?		X		
6. Was there clear reporting of the demographics of the participants in the study?		X		

7. Was there clear reporting of clinical information of the participants?			X	
8. Were the outcomes or follow up results of cases clearly reported?	X			
9. Was there clear reporting of the presenting site(s)/clinic(s) demographic information?		X		
10. Was statistical analysis appropriate?				X
**Matos et al. (2010)**				
**Questions**	**Yes**	**No**	**Unclear**	**Not applicable**
1. Were there clear criteria for inclusion in the case series?	X			
2. Was the condition measured in a standard, reliable way for all participants included in the case series?	X			
3. Were valid methods used for identification of the condition for all participants included in the case series?		X		
4. Did the case series have consecutive inclusion of participants?	X			
5. Did the case series have complete inclusion of participants?	X			
6. Was there clear reporting of the demographics of the participants in the study?		X		
7. Was there clear reporting of clinical information of the participants?	X			
8. Were the outcomes or follow up results of cases clearly reported?	X			
9. Was there clear reporting of the presenting site(s)/clinic(s) demographic information?		X		
10. Was statistical analysis appropriate?	X			
**Frazao e Manzi et al. (2012)**				
**Questions**	**Yes**	**No**	**Unclear**	**Not applicable**
1. Were there clear criteria for inclusion in the case series?	X			
2. Was the condition measured in a standard, reliable way for all participants included in the case series?	X			
3. Were valid methods used for identification of the condition for all participants included in the case series?		X		

4. Did the case series have consecutive inclusion of participants?		X		
5. Did the case series have complete inclusion of participants?		X		
6. Was there clear reporting of the demographics of the participants in the study?		X		
7. Was there clear reporting of clinical information of the participants?	X			
8. Were the outcomes or follow up results of cases clearly reported?	X			
9. Was there clear reporting of the presenting site(s)/clinic(s) demographic information?		X		
10. Was statistical analysis appropriate?				X
